# Adolescent and Early-Onset Adenomyosis: Phenotypes, Diagnostic Challenges, and Fertility-Conscious Care

**DOI:** 10.7150/ijms.140803

**Published:** 2026-10-02

**Authors:** Shuhong Yang, Yuling Chen, Xiangyi Ma

**Affiliations:** 1 Department of Obstetrics and Gynecology, Tongji Hospital, Tongji Medical College, Huazhong University of Science and Technology, Wuhan, Hubei, 430030, China.; 2 National Clinical Research Center for Obstetrical and Gynecological Diseases, Wuhan, Hubei, 430030, China.; 3 Key Laboratory of Cancer Invasion and Metastasis, Ministry of Education, Wuhan, Hubei, 430030, China.

**Keywords:** Adenomyosis, adolescence, dysmenorrhea, endometriosis, fertility

## Abstract

Adolescent and early-onset adenomyosis is increasingly recognized in symptomatic young patients, but its clinical significance remains uncertain. This narrative review synthesizes English-language literature on adolescent and early-onset adenomyosis, distinguishing direct adolescent and young-adult evidence from supportive evidence derived from adult populations. Current data suggest that adenomyosis-like imaging features may be identified in adolescents with severe dysmenorrhea, heavy menstrual bleeding, chronic pelvic pain, or coexisting endometriosis. Early disease often appears as subtle diffuse inner-myometrial change, focal lesions, or cystic/juvenile forms rather than the classic diffuse adult phenotype. Ultrasound is the first-line imaging modality, whereas MRI is reserved for equivocal findings, atypical cystic lesions, suspected congenital anomalies, or endometriosis overlap. Management requires a stepwise, symptom-oriented approach that considers future reproductive options and avoids unnecessary uterine intervention. Overall, adolescent adenomyosis should be considered in patients with severe or treatment-resistant menstrual symptoms, while uncertain imaging findings should not be overinterpreted.

## 1. Introduction

Severe dysmenorrhea, heavy menstrual bleeding, and chronic pelvic pain during adolescence are often attributed to functional menstrual symptoms or primary dysmenorrhea 1, 2. This interpretation may delay evaluation for underlying gynecological disease, particularly when symptoms are persistent, disabling, or poorly responsive to first-line analgesic treatment 3-5. In this context, adenomyosis has received increasing attention as a possible yet underrecognized contributor to severe menstrual symptoms in adolescents and young women 6.

Adenomyosis has traditionally been regarded as a disorder of later reproductive life, largely because earlier descriptions were based on hysterectomy specimens and adult surgical series. However, wider use of high-resolution pelvic ultrasound and magnetic resonance imaging has challenged this age-based concept. Imaging studies now suggest that adenomyosis-like features may be detected before adulthood or in early reproductive life, particularly in patients with severe dysmenorrhea, heavy menstrual bleeding, pelvic pain, or coexisting endometriosis. Young age alone should therefore not exclude adenomyosis from the differential diagnosis.

The clinical significance of adolescent adenomyosis remains uncertain. Unlike classic adult phenotype, early-onset adenomyosis may present as subtle junctional-zone abnormalities, focal inner-myometrial lesions, or cystic phenotypes rather than diffuse uterine enlargement. These findings may be overlooked when clinicians rely on the traditional adult template, but they may also be overinterpreted when isolated imaging signs are considered without symptom burden or developmental context. This diagnostic tension is particularly relevant in adolescents because age-specific imaging thresholds have not been established.

The evidence base is also heterogeneous. Many studies include both adolescents and young adult women, whereas much of the mechanistic and reproductive literature is derived from adult populations. It remains unclear whether adenomyosis recognized during adolescence represents an early stage of the adult disease spectrum, a distinct early-onset phenotype, or a heterogeneous group of conditions with different trajectories. Natural history, optimal diagnostic criteria, treatment response, and long-term reproductive implications remain poorly defined.

To address this heterogeneity, this review uses “adolescent adenomyosis” as an operational term for adenomyosis or adenomyosis-like imaging findings identified during adolescence, generally corresponding to patients aged 10-19 years according to the WHO definition of adolescence. Because no universally accepted age threshold exists for “early-onset adenomyosis,” we use this broader term to include adolescent cases and adenomyosis recognized in young women near the transition from adolescence to adulthood, particularly when symptoms or imaging features suggest onset early in reproductive life. Accordingly, evidence from cohorts spanning adolescence and early adulthood, such as patients aged 12-25 or 13-25 years, is interpreted as mixed adolescent/young-adult evidence, whereas studies in young nulligravid women aged up to 30 years are considered supportive young-adult evidence rather than strictly adolescent evidence.

This review provides a clinically oriented synthesis of adolescent and early-onset adenomyosis. We distinguish direct adolescent evidence from supportive inference derived from young adult and broader reproductive-age populations, and focus on epidemiologic signals, early phenotypes, diagnostic pitfalls, imaging interpretation, management principles, and fertility-conscious longitudinal care. Our aim is to support earlier recognition of clinically meaningful disease while avoiding overdiagnosis and inappropriate extrapolation from adult adenomyosis.

## 2. Literature identification strategy

This review was designed as a clinically oriented narrative review of adolescent and early-onset adenomyosis. PubMed, Embase, and Web of Science were searched for English-language articles published up to March 5, 2026. The search focused on four core concepts: adenomyosis; adolescence or early-onset disease; symptoms and comorbidity; and diagnosis, management, or reproductive implications. The database-specific search strategies are provided in Supplementary Table S1.

Articles were considered eligible if they provided clinically relevant information on adolescent or early-onset adenomyosis, including disease burden, symptoms, imaging phenotypes, adenomyosis-endometriosis overlap, differential diagnosis, management, follow-up, reproductive implications, or relevant mechanisms. Direct adolescent evidence was prioritized; mixed adolescent/young-adult studies were interpreted as supportive early-onset evidence, and adult studies were used only when adolescent-specific data were unavailable. After duplicate removal, titles and abstracts were screened by two authors, followed by full-text assessment of potentially relevant articles. Disagreements were resolved through discussion with the senior author. Supplementary Figure S1 summarizes the literature identification process but should not be interpreted as a PRISMA flow diagram; no formal risk-of-bias assessment or quantitative synthesis was performed.

## 3. Epidemiologic signals and early clinical phenotypes

### 3.1 Disease burden in symptomatic adolescents and young women

The population-level epidemiology of adolescent adenomyosis remains poorly defined. Most available estimates come from imaging-based studies of symptomatic adolescents and young women evaluated in referral settings. Reported prevalence figures should therefore be interpreted as indicators of disease burden in selected clinical populations rather than as estimates of adenomyosis prevalence in the general adolescent population.

Even with this limitation, current evidence suggests that adenomyosis is not uncommon among highly symptomatic young patients. In a cohort of 267 adolescents aged 12-20 years with severe dysmenorrhea, Exacoustos et al. reported ultrasonographic signs suggestive of endometriosis and/or adenomyosis in 106 patients (39.7%), with adenomyosis identified in 47 patients, corresponding to 17.6% of the total cohort 7. In a cross-sectional study of patients aged 13-25 years with dysmenorrhea and/or heavy menstrual bleeding, Vannuccini et al. identified adenomyosis in 27.4% of cases using Morphological Uterus Sonographic Assessment (MUSA) criteria, suggesting that symptom clustering may help identify early-onset disease 5.

Evidence syntheses support this signal while emphasizing heterogeneity. Vercellini et al. reported a weighted pooled adenomyosis prevalence of 20.7% (95% CI, 11.5-31.6; I²=94.8%) across six imaging-based studies in symptomatic adolescents and young women, with subgroup estimates of 16.9% (95% CI, 8.8-27.0; I²=91.2%) in adolescents and 29.7% (95% CI, 17.5-43.5; I²=85.6%) in young women. These data suggest that adenomyosis-related imaging findings are not uncommon in selected symptomatic populations, but they do not provide a reliable estimate of population prevalence in adolescents 8, 9. Earlier MRI-based evidence also suggested that adenomyosis-related junctional-zone changes may emerge relatively early in reproductive life, particularly in women with endometriosis, although these data were not derived from strictly adolescent cohorts 10. However, differences in diagnostic criteria, imaging expertise, symptom selection, and age grouping limit direct comparison across studies. Current data therefore do not establish the true prevalence of adolescent adenomyosis, but they do indicate a clinically meaningful disease signal in young patients with severe menstrual symptoms. The key studies informing current understanding are summarized in Table 1, and imaging-based prevalence studies are summarized in Supplementary Table S2.

### 3.2 Early, focal and cystic phenotypes

Early-onset adenomyosis appears to differ from the classic adult phenotype derived from hysterectomy-based and advanced surgical series. In adolescents and young women, adenomyosis is more often described as mild, focal, or cystic rather than as diffuse bulky myometrial involvement 12, 13, 18, 19. This distinction is clinically important because under-recognition in younger patients may reflect not only low clinical suspicion, but also a mismatch between early disease morphology and the traditional adult image of adenomyosis.

Among adolescent studies, adenomyosis is more commonly classified as mild or moderate, whereas severe diffuse disease appears relatively uncommon 12. Supportive evidence from young nulligravid women aged 18-30 years points in the same direction, suggesting that adenomyosis-like changes may emerge early in reproductive life and may already be associated with dysmenorrhea and heavier menstrual bleeding 20. Although such young adult data cannot be treated as adolescent-specific evidence, they provide transitional support for the concept that adenomyotic changes may precede the age range in which the disease has historically been diagnosed.

Phenotypically, adolescent and early-onset adenomyosis may be conceptualized as three broad patterns: subtle diffuse inner-myometrial change, focal inner-myometrial lesions, and cystic or juvenile adenomyotic lesions. This framework is useful because phenotypes may influence symptom profile, differential diagnosis, imaging interpretation, and treatment intensity 12, 13. Juvenile cystic adenomyoma and related cystic lesions may present with severe dysmenorrhea and disproportionate pain despite localized disease 13, 15-18.

The predominance of mild, focal, and cystic disease should not be interpreted as evidence that all early lesions inevitably progress to diffuse adult adenomyosis. Rather, current findings support a more cautious interpretation: adolescent adenomyosis likely represents a spectrum of early phenotypes with incompletely characterized trajectories. The main value of phenotype recognition lies in improving diagnostic accuracy, avoiding both under-recognition and overdiagnosis, and aligning management intensity with the likely burden of disease. The early phenotypic spectrum and its clinical implications are summarized in Figure 1.

### 3.3 Comorbidity and key differential diagnoses

Adenomyosis in adolescents frequently coexists with other gynecological disorders, most notably endometriosis 1, 7, 8, 21, 22. This overlap is clinically important because it may increase symptom severity, complicate imaging interpretation, and influence treatment planning. In adolescent and early-onset cohorts, severe dysmenorrhea, chronic pelvic pain, and suspected or confirmed endometriosis repeatedly emerge as the main clinical context in which adenomyosis is identified.

In one adolescent cohort reported by Exacoustos et al., approximately 44% of adolescents diagnosed with adenomyosis were found to have at least one concomitant pelvic endometriotic lesion 12. Likewise, Zannoni et al. reported a high burden of both adenomyosis and endometriosis in adolescents and young women with pelvic pain, with a significant association between ovarian endometriosis and adenomyosis 11**.** These findings suggest that the two disorders may share common pathogenic mechanisms or developmental pathways.

At the same time, diagnostic assessment in adolescents should remain attentive to important differential diagnoses. Accessory cavitated uterine malformation (ACUM), juvenile cystic adenomyoma, and congenital uterine anomalies may mimic adenomyosis on imaging and may also present with severe progressive dysmenorrhea 23-26. This is especially relevant in younger patients because cystic lesions and unusual uterine morphology may be over-attributed to adenomyosis if imaging is interpreted without adequate developmental and anatomic context.

For this reason, comorbidity assessment and differential diagnosis should be considered together in adolescent practice. The key clinical question is not simply whether adenomyosis is present, but whether the patient's symptoms and imaging findings are best explained by isolated adenomyosis, adenomyosis with endometriosis overlap, or an alternative structural condition requiring a different management pathway. This broader diagnostic frame is essential to avoid both under-recognition of overlap disease and misclassification of congenital or cystic lesions as adenomyosis.

## 4. Biological plausibility and mechanistic heterogeneity

The pathogenesis of adenomyosis remains incompletely understood and is likely multifactorial. Altered uterine peristalsis, tissue injury and repair, hormonal dysregulation, inflammation, and fibrotic remodeling may contribute to disease initiation and progression. Different anatomical phenotypes, including diffuse, focal, cystic, inner-myometrial, outer-myometrial, and endometriosis-associated disease, may therefore arise through partially distinct pathways rather than through a single uniform mechanism 27, 28.

Junctional-zone dysfunction and tissue injury-repair models provide one plausible explanation for early inner-myometrial disease, linking severe dysmenorrhea, uterine biomechanics, and subtle inner-myometrial or focal imaging changes 27, 28. Other proposed mechanisms, including metaplastic transformation, stem/progenitor-cell differentiation, and outside-to-inside extension in endometriosis-associated phenotypes, may help explain focal, cystic, outer-myometrial, or overlap disease 28-30.

However, the adolescent relevance of these mechanisms remains inferential. Most mechanistic and molecular studies are based on adult tissue or surgical populations with established disease. Therefore, current pathophysiological models should be interpreted as biologically plausible explanations for early-onset phenotypes rather than as adolescent-specific evidence.

## 5. Diagnosis in adolescents: from clinical suspicion to imaging interpretation

The diagnosis of adenomyosis has shifted from postoperative histopathological confirmation to non-invasive recognition based on symptoms and imaging. This shift is particularly relevant in adolescents and young women, in whom earlier identification may improve symptom control and reduce delayed recognition of underlying gynecological disease. At the same time, diagnosis in this age group remains challenging because menstrual pain is often normalized, imaging findings may be subtle, and age-specific diagnostic thresholds have not been fully established. Diagnostic evaluation should therefore be regarded as a structured, risk-based process rather than a single imaging event. Symptom severity, response to first-line treatment, suspected endometriosis overlap, and imaging phenotype all contribute to diagnostic confidence.

### 5.1 Clinical red flags

The most important clinical clue is severe dysmenorrhea. In adolescent cohorts with ultrasound-diagnosed adenomyosis, dysmenorrhea is reported in most patients, often in highly symptomatic referral settings 8, 12. Imaging findings suggestive of endometriosis and/or adenomyosis have also been identified in a substantial proportion of adolescents with severe dysmenorrhea, indicating that marked menstrual pain should not automatically be regarded as functional or “normal” 7, 31.

Heavy menstrual bleeding is another important signal. In symptomatic adolescents and young women, adenomyosis appears to be identified more often when dysmenorrhea and heavy menstrual bleeding coexist than when pain is considered alone 5, 32. Other features that should prompt earlier evaluation include poor response to first-line analgesics, chronic pelvic pain outside menstruation, recurrent school or activity impairment, and symptoms suggestive of coexisting endometriosis. Family history may provide additional context, but current evidence is indirect and should not be treated as diagnostic 22, 33, 34. In practice, it is the pattern of severe, persistent, treatment-resistant symptoms—especially when accompanied by heavy menstrual bleeding or chronic pelvic pain—that should trigger targeted imaging assessment. The main clinical red flags prompting targeted evaluation are summarized in Figure 2.

### 5.2 Ultrasound, MRI, and phenotype-aware interpretation

Ultrasound should remain the first-line imaging modality for suspected adolescent adenomyosis because it is accessible, repeatable, and capable of identifying subtle or focal myometrial abnormalities when performed by experienced operators 35-37. Its value in adolescents lies not only in detecting diffuse disease, but also in recognizing subtle inner-myometrial changes, focal lesions, and cystic phenotypes that may be more typical of early-onset disease 5, 12. The ultrasound route should also be individualized. Transabdominal ultrasound is usually the initial approach, particularly in younger or sexually inactive patients, whereas transvaginal, transrectal, or transperineal approaches may be considered selectively according to sexual history, patient acceptability, local practice, and operator expertise 38.

According to the revised Morphological Uterus Sonographic Assessment (MUSA) consensus, direct features include myometrial cysts, hyperechogenic islands, and echogenic subendometrial lines or buds, whereas indirect features include globular uterus, asymmetrical myometrial thickening, fan-shaped shadowing, translesional vascularity, and an irregular or interrupted junctional zone 39. Because diagnostic performance remains operator-dependent, standardized acquisition and reporting are essential 36, 40. In adolescents, direct features or multiple concordant findings in a highly symptomatic patient may increase diagnostic confidence, whereas isolated indirect or equivocal findings should prompt cautious interpretation, expert review, or interval reassessment.

MRI has a complementary role when ultrasound findings are inconclusive, lesion phenotype is atypical, or the differential diagnosis includes congenital anomalies, cystic lesions, or deep infiltrating endometriosis 37, 41-43. Typical MRI features include junctional-zone thickening or irregularity, poorly defined low-signal-intensity myometrial areas on T2-weighted imaging, and cystic change in selected phenotypes. In adolescents, MRI is most useful when severe pelvic pain persists despite non-diagnostic ultrasound, when outer-myometrial disease or endometriosis overlap is suspected, or when differentiation from congenital uterine anomalies is required 44, 45. However, MRI should not be used as a routine second-line test for all adolescents with dysmenorrhea. Cost, access, and the lack of robust age-specific thresholds limit its role as a screening modality. It is best used as a targeted problem-solving tool.

### 5.3 Emerging adjuncts, diagnostic pitfalls, and overdiagnosis

Elastography, artificial intelligence, and radiomics may eventually refine image-based assessment of adenomyosis, but they should currently be regarded as investigational adjuncts rather than routine tools in adolescent diagnostic pathways. Elastography is biologically plausible because adenomyosis is associated with fibrosis and myometrial remodeling, and adult studies suggest that tissue stiffness may help distinguish adenomyosis from normal myometrium or correlate with symptom burden 46-49. Similarly, artificial intelligence and radiomics have shown promise in adult ultrasound and MRI datasets, but most models have been developed in tertiary-care populations with established disease, and their thresholds, generalizability, and clinical value in adolescents remain uncertain 50, 51.

Earlier recognition also increases the risk of overdiagnosis. In adolescents, this risk is especially important because the uterus is still developing, imaging findings may be subtle or nonspecific, and thresholds for pathologic versus physiologic myometrial variation remain insufficiently defined. One major pitfall is over-attributing severe dysmenorrhea to adenomyosis based on isolated indirect or equivocal imaging findings. The opposite problem is failure to recognize early disease because it does not resemble the classic diffuse adult phenotype. A balanced diagnostic strategy therefore requires correlation between symptom severity, imaging phenotype, and differential diagnosis rather than reliance on a single sign or label.

Interobserver variability remains another challenge. Even with revised MUSA terminology, reproducibility of some ultrasound features is imperfect, highlighting the need for standardized acquisition, reporting, and reader training 39, 52. Differential diagnosis must also remain broad, particularly in adolescents with severe progressive dysmenorrhea and atypical cystic or structural findings. Overall, current evidence supports a risk-based diagnostic model: adolescents with severe symptoms, direct MUSA features, multiple concordant imaging features, or overlap disease warrant closer evaluation and follow-up, whereas mild symptoms with isolated or equivocal findings may justify observation and reassessment. This approach acknowledges both the clinical importance of adolescent adenomyosis and the uncertainty surrounding its earliest imaging manifestations.

## 6. Management of adolescent adenomyosis: symptom-oriented longitudinal care with attention to future reproductive options

Management of adolescent adenomyosis should be individualized and proportionate. Unlike management in older adults, treatment in adolescents must address symptom relief, bleeding control, long-term adherence, avoidance of overtreatment, and preservation of future reproductive options. Because adolescent-specific therapeutic evidence remains limited, management should be guided by symptom burden, imaging phenotype, suspected overlap disease, treatment response, and patient priorities, with cautious extrapolation from adult adenomyosis, adolescent dysmenorrhea or endometriosis, and broader chronic pelvic pain care.

### 6.1 Stepwise medical management

For most adolescents, initial treatment is conservative and symptom-oriented. Non-steroidal anti-inflammatory drugs (NSAIDs) are appropriate first-line therapy for mild or moderate dysmenorrhea, as recommended in adolescent dysmenorrhea guidance 53, 54. This approach is especially reasonable when imaging findings are uncertain or when symptoms remain manageable without long-term suppression.

When symptoms are recurrent, severe, or insufficiently controlled, hormonal suppression becomes the main therapeutic option. Combined hormonal contraceptives and progestins are commonly used on the basis of adolescent dysmenorrhea/endometriosis guidance and extrapolation from adult adenomyosis management, whereas gonadotropin-releasing hormone agonists should be reserved for selected refractory cases because of bone-health concerns and the need for add-back therapy 53, 55, 56. Small adolescent case-series evidence also suggests that hormonal suppression may reduce MRI-visible adenomyosis in selected patients, but this remains low-level evidence 14.

Treatment selection should also consider tolerability, bleeding pattern, expected duration of use, adherence, contraceptive needs, bone health, and endocrine maturation. Medical therapy should therefore be presented as an adjustable longitudinal strategy rather than as a fixed long-term solution for all patients.

### 6.2 Refractory or complex disease

Persistent symptoms despite appropriate first-line treatment should prompt reassessment rather than automatic escalation. In adolescents, refractory symptoms may reflect coexisting endometriosis, pelvic floor dysfunction, central pain amplification, poor adherence, treatment intolerance, or an alternative structural diagnosis. Treatment response should therefore be interpreted cautiously: improvement in bleeding or cyclic dysmenorrhea after hormonal suppression may reflect suppression of endometrial, adenomyotic, or endometriotic activity, whereas persistent acyclic pelvic pain, bowel or urinary symptoms, or functional impairment should raise suspicion of coexisting endometriosis or mixed pain mechanisms. Recent longitudinal evidence on adenomyosis with coexisting endometriosis also supports individualized follow-up according to symptom evolution, imaging phenotype, treatment exposure, and reproductive priorities 57. Management should therefore include review of the original diagnosis, reassessment of symptom pattern, expert imaging review when needed, and evaluation for overlap disease 2.

For patients with disabling pain, recurrent functional impairment, or suspected mixed pain mechanisms, management may extend beyond uterine-directed therapy to include pelvic floor physical therapy, pain education, psychological or behavioral support, and multidisciplinary pain care, consistent with broader chronic pelvic pain guidance 58. The key principle is that refractory adolescent adenomyosis should not be managed solely as a uterine imaging abnormality; pain mechanisms and functional consequences also need attention.

### 6.3 Selected intervention and longitudinal follow-up

Surgical or interventional treatment should be reserved for carefully selected adolescents with focal cystic lesions or juvenile cystic adenomyoma refractory to medical treatment, as supporting evidence is limited to case reports and small case series 18. In such cases, uterus-sparing laparoscopic excision may be considered when severe symptoms persist despite appropriate medical therapy and when expert imaging identifies a discrete lesion. Potential risks, including uterine scarring, recurrence, and uncertain reproductive consequences, should be discussed before intervention.

High-intensity focused ultrasound and other non-invasive techniques have been explored as fertility-preserving approaches in adenomyosis, but adolescent evidence remains very limited. These modalities should currently be regarded as selective or investigational rather than routine care for young patients 59.

Longitudinal follow-up is central to adolescent care. Symptoms, menstrual pattern, treatment tolerance, imaging phenotype, overlap disease, and reproductive priorities may all change over time. Follow-up should therefore include planned reassessment rather than a one-time treatment decision. During transition to adult care, patients with suspected or confirmed adenomyosis should ideally have a clear summary of symptom history, imaging findings, prior treatments, treatment response, and unresolved concerns. This is particularly important for those with persistent pain, repeated imaging abnormalities, suspected endometriosis overlap, or future fertility concerns.

Overall, current evidence supports a stepwise and fertility-conscious model: medical treatment for most symptomatic adolescents, broader multidisciplinary assessment for refractory or complex disease, and invasive treatment only for selected focal or cystic lesions. This approach balances symptom control with caution, avoids unnecessary intervention, and preserves future reproductive options. A symptom- and phenotype-based diagnostic and management pathway for suspected adolescent adenomyosis is summarized in Figure 3.

## 7. Discussion

Adolescent and early-onset adenomyosis is best approached as a clinically relevant but still incompletely defined condition. The available evidence supports considering adenomyosis in adolescents and young women with severe or treatment-resistant dysmenorrhea, heavy menstrual bleeding, chronic pelvic pain, or coexisting endometriosis. At the same time, early imaging findings cannot be interpreted in isolation. Their clinical meaning depends on symptom burden, phenotype, developmental context, and alternative diagnoses.

This distinction is important in daily practice. Some adolescents with persistent symptoms may be under-investigated because adenomyosis is still perceived as a disease of older reproductive age. Others may be overdiagnosed when subtle or equivocal myometrial findings are treated as definitive disease. A risk-based approach is therefore more appropriate than a simple positive-or-negative diagnostic label. Multiple concordant imaging features in a highly symptomatic patient carry different clinical weight from an isolated finding in a mildly symptomatic adolescent.

Fertility counselling is another area where caution is essential. Adult studies have linked adenomyosis with infertility, implantation failure, poorer assisted-reproduction outcomes, and adverse obstetric events, particularly in patients with endometriosis overlap or specific imaging phenotypes 60-62. Different phenotypes may also carry different reproductive implications, although this evidence is mainly derived from adult, infertility, or assisted-reproduction populations 63. Available studies suggest that symptomatic and diffuse adenomyosis may be more strongly associated with reduced clinical pregnancy or live-birth rates and increased miscarriage risk after in vitro fertilization than asymptomatic adenomyosis 64. Recent subtype-based evidence also suggests that internal or mixed adenomyosis may be associated with lower live-birth rates and higher early pregnancy loss after frozen embryo transfer, whereas external adenomyosis may have less apparent impact 65. Disease burden and lesion localization may therefore be relevant when counselling young patients, particularly those with progressive imaging findings, coexisting endometriosis, or future fertility concerns 66, 67. However, these observations should not be directly extrapolated to adolescents; instead, they support cautious counselling, avoidance of unnecessary uterine intervention, and longitudinal documentation of symptoms, imaging findings, and treatment response.

A strength of this review is that it separates direct adolescent evidence from evidence extrapolated from young adult or broader adult populations. This distinction matters because applying adult adenomyosis data too directly to adolescents may exaggerate diagnostic certainty, treatment implications, or reproductive risk. The review also brings together epidemiologic signals, early phenotypes, biological plausibility, diagnostic challenges, management principles, and fertility-conscious care within a life-course framework.

The limitations mainly reflect the state of the literature. Most studies are cross-sectional, imaging-based, and drawn from symptomatic or referral populations. Adolescents are often analyzed together with young adult women, making it difficult to define true prevalence, natural history, and phenotype-specific outcomes in strictly adolescent cohorts. Treatment evidence is limited, and most management strategies remain extrapolated from adult adenomyosis, adolescent dysmenorrhea, endometriosis, or chronic pelvic pain care. Mechanistic and reproductive data are also largely adult-derived.

Future studies should move beyond earlier detection alone. Prospective multicenter cohorts with standardized symptom assessment, ultrasound and MRI reporting, and long-term follow-up are needed to determine which early lesions are clinically meaningful. Age-conscious diagnostic criteria may help distinguish disease from normal developmental variation or equivocal myometrial findings. Further work should also clarify whether focal, diffuse, cystic, inner-myometrial, outer-myometrial, or endometriosis-associated phenotypes differ in symptom persistence, treatment escalation, progression, and reproductive outcomes.

Overall, the key question is not simply whether adenomyosis can be detected earlier, but which adolescents require closer follow-up and which management strategies improve long-term pain control, bleeding patterns, quality of life, and reproductive outcomes.

## Supplementary Material

Supplementary figure and tables.

## Figures and Tables

**Figure 1:**
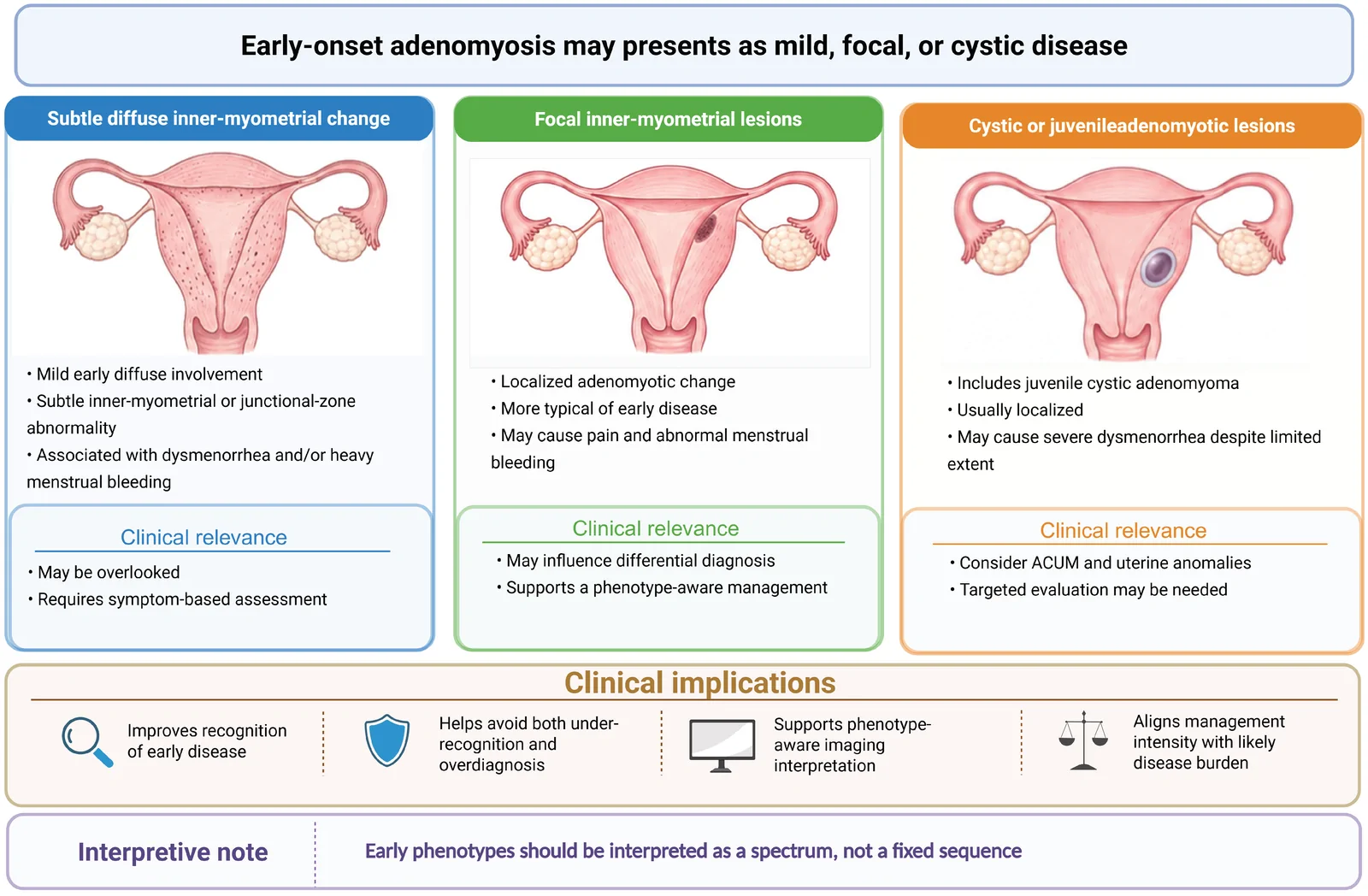
Early phenotypic spectrum and clinical implications of adolescent adenomyosis. ACUM, accessory cavitated uterine malformation. Created with BioRender.com.

**Figure 2:**
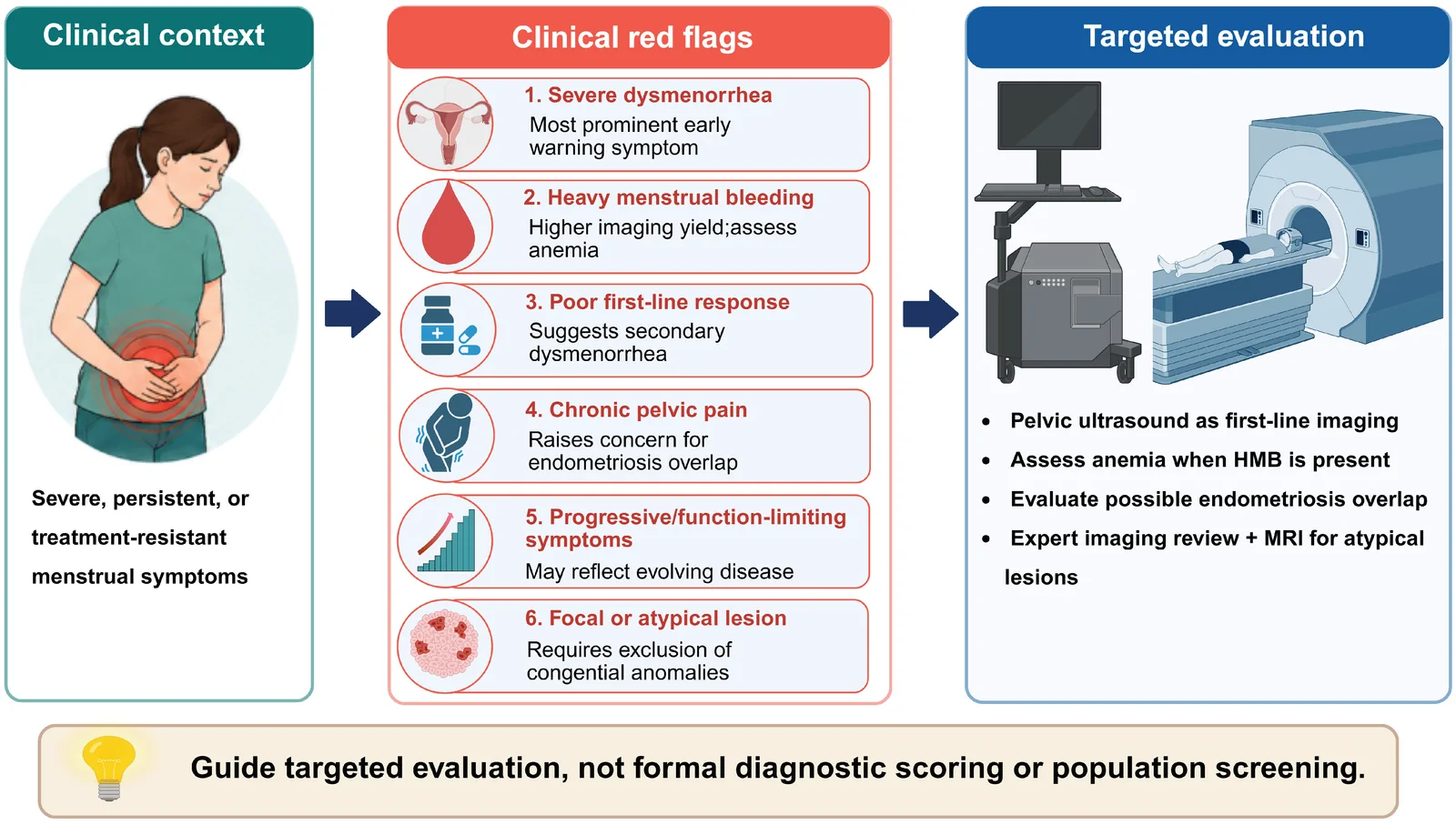
Clinical red flags prompting targeted evaluation of suspected adolescent adenomyosis.** Abbreviations:** HMB, heavy menstrual bleeding; MRI, magnetic resonance imaging. Created with BioRender.com.

**Figure 3:**
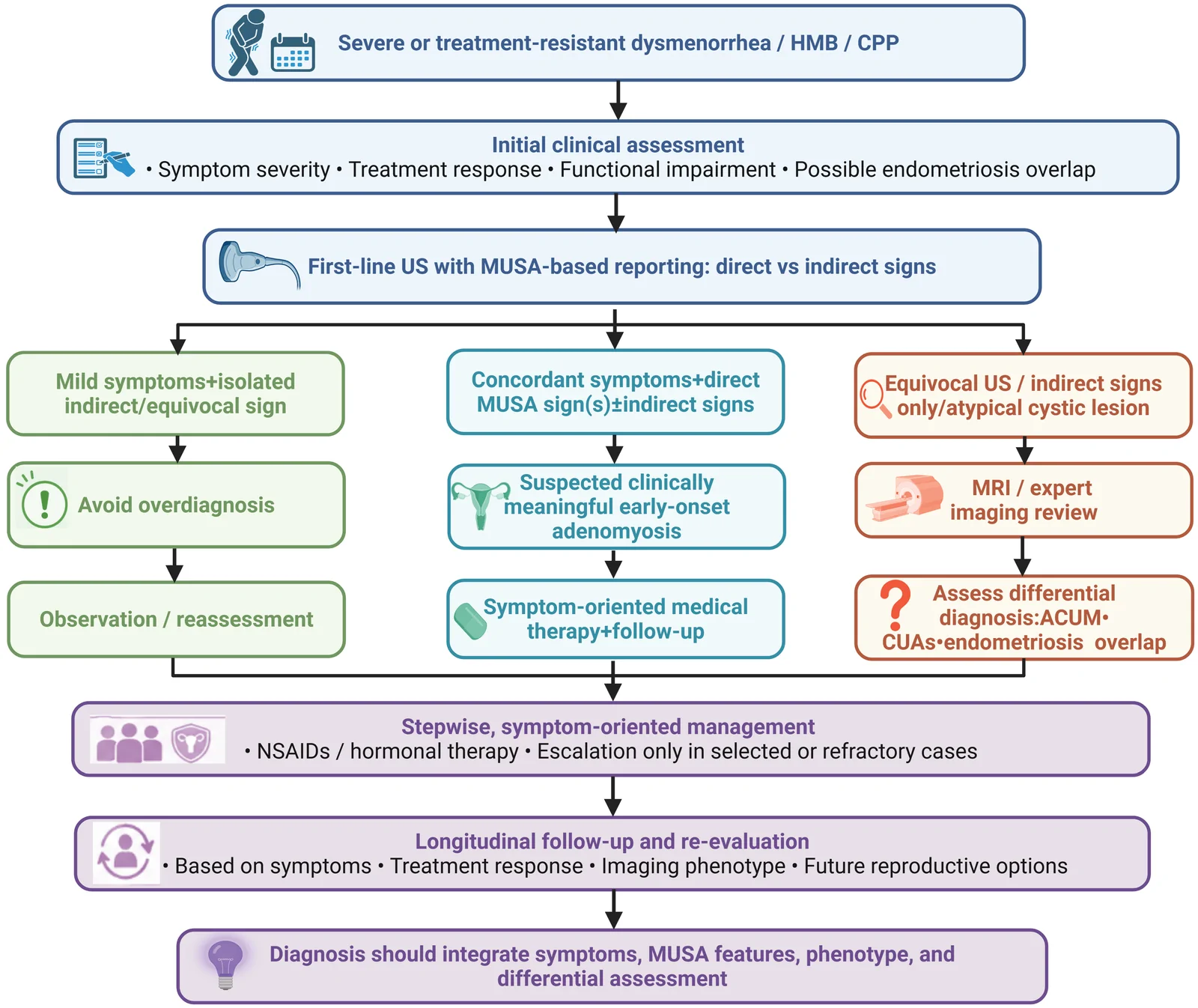
** Symptom- and phenotype-based diagnostic and management pathway for suspected adolescent or early-onset adenomyosis.** Direct and indirect MUSA features should be interpreted in clinical context, because isolated indirect or equivocal findings may be nonspecific in adolescents. Abbreviations: ACUM, accessory cavitated uterine malformation; CPP, chronic pelvic pain; CUAs, congenital uterine anomalies; HMB, heavy menstrual bleeding; MRI, magnetic resonance imaging; MUSA, Morphological Uterus Sonographic Assessment; NSAIDs, non-steroidal anti-inflammatory drugs; US, ultrasound. Created with BioRender.com.

**Table 1:** Key studies on adolescent and early-onset adenomyosis.

Study	Evidence source/Population	Diagnostic modality	Key contribution
Exacoustos et al. 2025 7	Direct adolescent cohort; 267 adolescents with severe dysmenorrhea	Ultrasound	Adenomyosis was identified in 47 of 267 adolescents with severe dysmenorrhea, supporting targeted ultrasound evaluation in highly symptomatic patients.
Wilk et al. 2025 8	Review-level adolescent evidence	Literature review of adolescent studies	Summarized prevalence, symptoms, and diagnostic pathways of adolescent adenomyosis and reinforced that the disease is likely underrecognized
Vercellini et al. 2025 9	Mixed adolescent/young-adult evidence synthesis	Imaging-based studies	Reported a weighted pooled prevalence of 20.7% with high heterogeneity, supporting a disease-burden signal in selected symptomatic adolescents and young women.
Martire et al. 2024 1	Review-level adolescent supportive evidence	Literature review	Framed adolescent adenomyosis as a challenging diagnosis with complex management and highlighted the need for age-specific approaches
Vannuccini et al. 2024 5	Mixed adolescent/young-adult evidence	Ultrasound using MUSA criteria	Demonstrated that adenomyosis can be identified in symptomatic adolescents/young women, particularly when dysmenorrhea and heavy menstrual bleeding coexist
Zannoni et al. 2024 11	Mixed adolescent/young-adult study of patients with pelvic pain	Clinical and imaging-based assessment	Highlighted adenomyosis-endometriosis overlap in young patients with pelvic pain.
Exacoustos et al. 2022 12	43 adolescents aged 12-20 years with ultrasonographically diagnosed adenomyosis	Ultrasound	Adenomyosis was predominantly mild to moderate, with no severe cases identified; the outer myometrium and posterior uterine wall were the most commonly affected sites.
Brosens et al. 2015 13	Supportive evidence on cystic adenomyosis in younger women	Imaging and pathology literature	Described uterine cystic adenomyosis as a phenotype mainly affecting younger women.
Mansouri et al. 2015 14	Adolescent case series	MRI before and after hormonal suppression	Reported regression of MRI-visible adenomyosis after hormonal suppression in adolescents
Ho et al. 2008^15^; Ho et al. 2009 16; Branquinho et al. 2012 17	Adolescent case reports	Ultrasound, MRI, and/or pathology	Reported adenomyotic cysts or juvenile cystic adenomyoma in adolescent girls, indicating that early disease may present as focal or cystic lesions
Dietrich 2010 4	Early narrative-review supportive evidence	Literature review	Summarized the early evidence on adolescent adenomyosis and emphasized diagnostic difficulty in teenagers
Takeuchi et al. 2010 18	Juvenile cystic adenomyoma case series; 9 cases	Imaging, laparoscopy, histopathology	Described diagnosis, laparoscopic treatment, and pathology of juvenile cystic adenomyoma
Itam et al. 2009 3	Adolescent case report and literature review	Imaging and clinical diagnosis	One of the earliest reports highlighting that adenomyosis can occur during adolescence and may present with severe dysmenorrhea

**Notes:** Evidence source/population indicates whether each study provides direct adolescent evidence, mixed adolescent/young-adult evidence, review-level evidence, or case-based supportive evidence. Case reports and case series were included to illustrate early focal or cystic phenotypes, not to estimate prevalence. **Abbreviations:** MRI, magnetic resonance imaging; MUSA, Morphological Uterus Sonographic Assessment.
