# Supplementary material for: Adolescent and Early-Onset Adenomyosis: Phenotypes, Diagnostic Challenges, and Fertility-Conscious Care
Source: Int J Med Sci. 2026 Oct 2;23(11):3453–63. doi: 10.7150/ijms.140803 (PMC13685038; doi:10.7150/ijms.140803)
Supplement: Supplementary file 1 [file ijmsv23p3453s1.pdf]

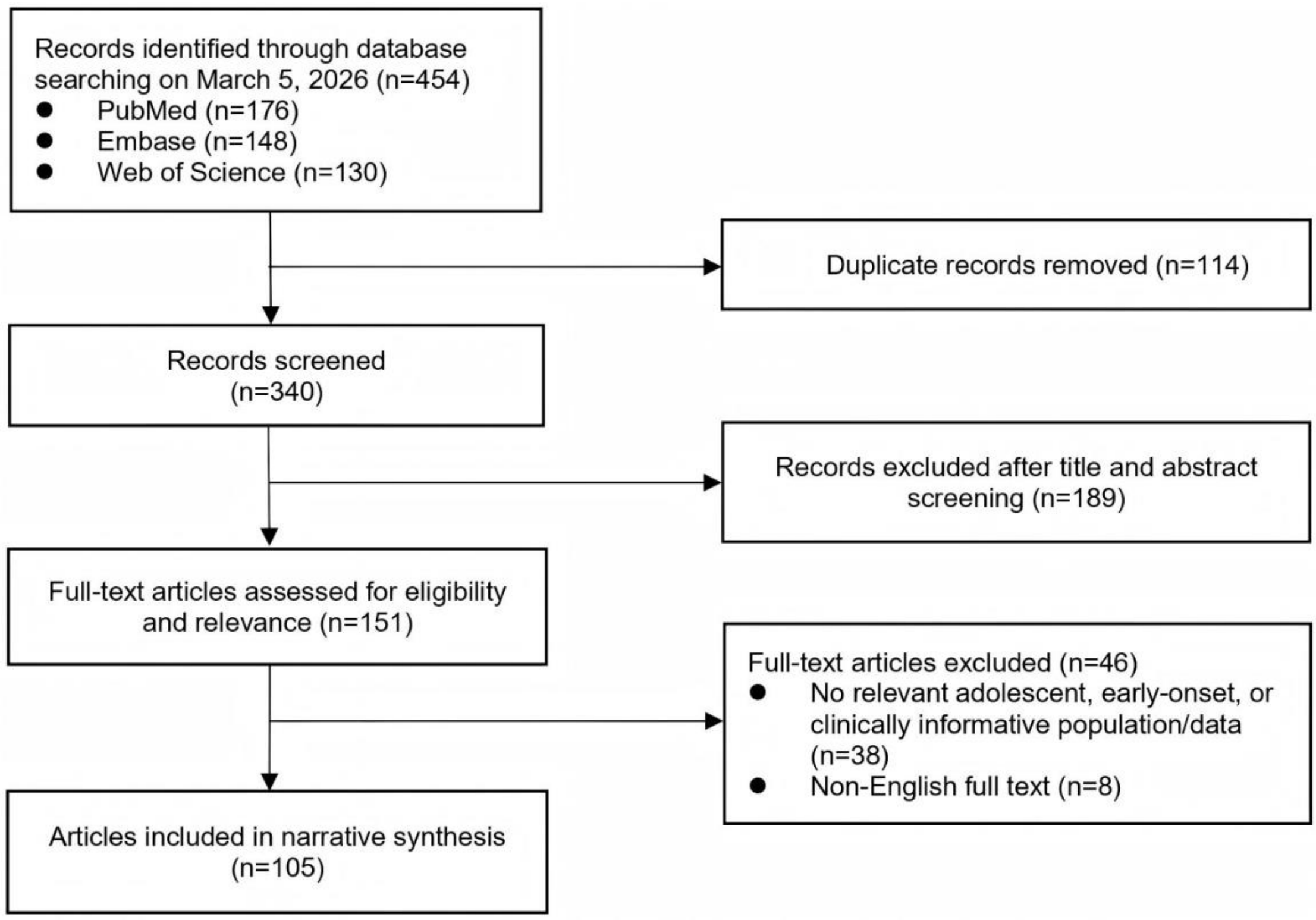

Supplementary Figure S1. Schematic overview of literature identification and evidence categorization for this narrative review. Relevant studies were identified through structured searches of PubMed, Embase, and Web of Science. Included literature was interpreted according to evidence source, including direct adolescent evidence, mixed adolescent/young-adult evidence, and supportive evidence from broader adult adenomyosis populations. Because this article is an evidence-informed narrative review rather than a formal systematic review, the figure should be interpreted as a conceptual overview of literature identification and evidence categorization rather than as a PRISMA flow diagram.

Supplementary Table S1. Database-specific search strategy.

Database	Search strategy
PubMed	(“adenomyosis” OR “adenomyoma” OR “cystic adenomyosis” OR “juvenile cystic adenomyoma” OR “accessory cavitated uterine malformation” OR “ACUM”) AND (“adolescent” OR “adolescence” OR “teenager” OR “young women” OR “early-onset” OR “early onset” OR “nulligravid”) AND (“dysmenorrhea” OR “heavy menstrual bleeding” OR

Database	Search strategy
	"chronic pelvic pain" OR "endometriosis" OR "ultrasound" OR "sonography" OR "MRI" OR "magnetic resonance imaging" OR "fertility" OR "pregnancy" OR "management" OR "treatment")
Embase	('adenomyosis' OR 'adenomyoma' OR 'cystic adenomyosis' OR 'juvenile cystic adenomyoma' OR 'accessory cavitated uterine malformation' OR 'ACUM') AND ('adolescent' OR 'adolescence' OR 'teenager' OR 'young women' OR 'early-onset' OR 'early onset' OR 'nulligravid') AND ('dysmenorrhea' OR 'heavy menstrual bleeding' OR 'chronic pelvic pain' OR 'endometriosis' OR 'ultrasound' OR 'sonography' OR 'MRI' OR 'magnetic resonance imaging' OR 'fertility' OR 'pregnancy' OR 'management' OR 'treatment')
Web of Science	TS=(adenomyosis OR adenomyoma OR "cystic adenomyosis" OR "juvenile cystic adenomyoma" OR "accessory cavitated uterine malformation" OR ACUM) AND TS=(adolescent OR adolescence OR teenager OR "young women" OR "early-onset" OR "early onset" OR nulligravid) AND TS=(dysmenorrhea OR "heavy menstrual bleeding" OR "chronic pelvic pain" OR endometriosis OR ultrasound OR sonography OR MRI OR "magnetic resonance imaging" OR fertility OR pregnancy OR management OR treatment)

Supplementary Table S2. Imaging-based prevalence estimates of adenomyosis in symptomatic adolescents and young women. Summary of estimates from symptomatic or referral-based cohorts. These figures should be interpreted as selected clinical-cohort estimates rather than population-level prevalence.

Study	Evidence source / population	Population	Clinical setting	Imaging modality	Sample size	Adenomyosis estimate / burden	Key interpretation
Exacoustos et al.(1)	Direct adolescent cohort; adolescents aged	Adolescents with severe dysmenorrhea	Symptomatic referral cohort	Ultrasound	267	Adenomyosis: 47/267 (17.6%);	Strong direct adolescent cohort; reflects imaging

Study	Evidence source / population	Population	Clinical setting	Imaging modality	Sample size	Adenomyosis estimate / burden	Key interpretation
	12–20 years with severe dysmenorrhea					composite endometriosis and/or adenomyosis findings: 106/267 (39.7%)	burden in highly symptomatic adolescents rather than population prevalence
Vannuccini et al.(2)	Mixed adolescent/young-adult cohort; patients aged 13–25 years with dysmenorrhea and/or heavy menstrual bleeding	Adolescents and young women aged 13–25 years with dysmenorrhea and/or heavy menstrual bleeding	Symptomatic clinical cohort	Ultrasound (MUSA criteria)	95	Adenomyosis diagnosed in 27.4% of patients	Key early-onset prevalence study linking pain plus heavy menstrual bleeding with adenomyosis detection
Vercellini et al.(3)	Evidence synthesis of symptomatic adolescents and young women with severe dysmenorrhea and/or heavy menstrual bleeding	Symptomatic adolescents and young women	Systematic review and meta-analysis of imaging-based studies	Ultrasound and/or MRI	6 studies; final quantitative population 1,274	Weighted pooled prevalence: 20.7% (95% CI, 11.5–31.6; I^2 = 94.8%); adolescent subgroup: 16.9% (95% CI, 8.8–27.0); young-women subgroup: 29.7% (95%	Best current pooled summary; substantial between-study heterogeneity should be acknowledged

Study	Evidence source / population	Population	Clinical setting	Imaging modality	Sample size	Adenomyosis estimate / burden	Key interpretation
						CI, 17.5–43.5)	

Abbreviations: CI, confidence interval; HMB, heavy menstrual bleeding; MUSA, Morphological Uterus Sonographic Assessment.

Notes:

1. Most currently available estimates derive from symptomatic or referral-based cohorts and are not population-based.
2. In Exacoustos et al.(1), 39.7% refers to the composite outcome of endometriosis and/or adenomyosis, whereas the adenomyosis-specific estimate is 47/267 (17.6%).
3. Kunz et al. (4) and Pinzauti et al.(5) were not included in this table because they provide supportive early reproductive-age imaging evidence but not directly comparable adolescent prevalence estimates in the current manuscript.

1. Exacoustos C, Mandosi C, Nocita E, Selntigia A, Iacobini F, Monaco G, et al. Severe dysmenorrhea in adolescents need non-invasive ultrasound evaluation to early detect endometriosis/adenomyosis. Eur J Obstet Gynecol Reprod Biol. 2025;313:114639.

2. Vannuccini S, Meleca C, Toscano F, Mertino P, Pampaloni F, Fambrini M, et al. Adenomyosis diagnosis among adolescents and young women with dysmenorrhoea and heavy menstrual bleeding. Reprod Biomed Online. 2024;48(5):103768.

3. Vercellini P BC, Bandini V, Cipriani S, Chiaffarino F, Viganò P, Somigliana E. Prevalence of adenomyosis in symptomatic adolescents and young women: a systematic review and meta-analysis. F S Rev. 2025;6:100083.

4. Kunz G, Herbertz M, Beil D, Huppert P, Leyendecker G. Adenomyosis as a disorder of the early and late human reproductive period. Reprod Biomed Online.

2007;15(6):681-5.

5. Pinzauti S, Lazzeri L, Tosti C, Centini G, Orlandini C, Luisi S, et al. Transvaginal sonographic features of diffuse adenomyosis in 18-30-year-old nulligravid women without endometriosis: association with symptoms. Ultrasound Obstet Gynecol. 2015;46(6):730-6.
